# Interaction of oxalate with β-glucan: Implications for the fungal extracellular matrix, and metabolite transport

**DOI:** 10.1016/j.isci.2023.106851

**Published:** 2023-05-10

**Authors:** Gabriel Perez-Gonzalez, Geoffrey A. Tompsett, Kyle Mastalerz, Michael T. Timko, Barry Goodell

**Affiliations:** 1 Department of Microbiology, University of Massachusetts, Amherst, MA 01003, USA; 2 Department of Chemical Engineering, Worcester Polytechnic Institute, 100 Institute Road, Worcester, MA 01609, USA

**Keywords:** Mycology, Cell biology, Microbial physics

## Abstract

β-glucan is the major component of the extracellular matrix (ECM) of many fungi, including wood degrading fungi. Many of these species also secrete oxalate into the ECM. Our research demonstrates that β-glucan forms a novel, previously unreported, hydrogel at room temperature with oxalate. Oxalate was found to alter the rheometric properties of the β-glucan hydrogels, and modeling showed that β-glucan hydrogen bonds with oxalate in a non-covalent matrix. Change of oxalate concentration also impacted the diffusion of a high-molecular-weight protein through the gels. This finding has relevance to the diffusion of extracellular enzymes into substrates and helps to explain why some types of wood-decay fungi rely on non-enzymatic degradation schemes for carbon cycling. Further, this research has potential impact on the diffusion of metabolites in association with pathogenic/biomedical fungi.

## Highlights

• β-glucan binds oxalate to form a hydrogel, a previously unreported finding
• Fungal ECM simulations showed that ECM hydrogels greatly limited protein diffusion
• Low-molecular-weight catecholates passed readily through high-oxalate hydrogels
• Microbial ECMs may impact biofilm formation, degradation mechanisms, and drug diffusion

## Graphical abstract

**Figure:**
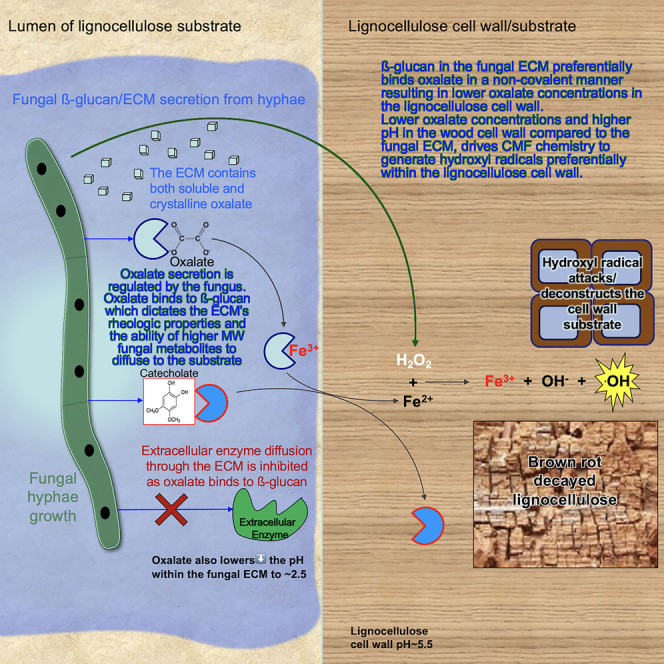

For a Figure360 author presentation of this figure, see https://doi.org/10.1016/j.isci.2023.106851.

## Introduction

Wood-degrading fungi govern the decomposition of the largest terrestrial reservoir of fixed carbon on the planet.^1^^,^^2^ Thus, these fungi are critical in carbon cycling in the environment, and potentially in regulating climate change.^3^ Brown rot (BR) wood-decay fungi are some of the most important species of decay fungi, particularly in the northern hemisphere where the BR fungi dominate lignocellulose degradation causing 80% or more of woody biomass degradation.^4^^,^^5^^,^^6^ These fungi also have potential use in the bioprocessing of woody materials for industrial applications including the generation of green platform chemicals and biofuels.^7^^,^^8^^,^^9^ However, many details are not understood relating to the mechanisms that both white rot (WR) and BR fungi use both to digest and to metabolize wood.

The extracellular matrix (ECM) is ubiquitous in microorganisms, and it surrounds the growing tips of virtually all BR and WR fungal hyphae^10^^,^^11^^,^^12^ where it plays a crucial role as the interface between the fungus and the woody substrate. The ECM consists largely of polysaccharides and functions in the attachment of fungal hyphae to substrates, as a transport medium regulating the movement of metabolites into the substrate and also as the interface allowing nutrients/water to pass from substrates to the hyphae.^11^ The ECM can also play a protective role in scavenging of reactive oxygen species^12^ or otherwise buffering microorganisms from environmental stress. Although there is a lack of information even about how the ECM is formed and functions in these fungi, the ECM does play a critical role in the efficiency with which BR and WR fungi process biomass. To better understand carbon cycling from terrestrial carbon sources as well as developing more efficient processes for the bioconversion of woody biomass for industrial applications, examining how the fungal ECM functions in decay fungi is of utmost importance. More broadly, understanding how fungal metabolites diffuse into substrates will provide greater understanding of the role of fungi in systems ranging from the environment to biomedical pathologies.

The fungal cell wall consists of chitin, glucans, and glycoproteins^13^^,^^14^^,^^15^ where chitin is layered adjacent to the fungal cell membrane and the glucan is then layered over the chitin. A surface glycoprotein layer then intergrades with the external fungal ECM. The fungal ECM generally consists of an array of polysaccharides, glycoproteins, and glycolipids,^11^ with most fungal ECMs including those from fungi that decay wood, generated with 1,3 β-glucan as their primary component.^16^^,^^17^^,^^18^^,^^19^ In our current research we have used 1,3/1,6 β-glucan from fungi (*Saccharomyces*), but it is recognized that the degree of 1,6 branching is known to vary with fungal species. In this paper we use “β-glucan” to refer generally to 1,3 β-glucan recognizing that branching patterns may vary with fungal species.

Bacterial ECMs have in some cases been reported to have channels that function in the transport of water and cellular materials between cells.^20^^,^^21^ At least some Basidiomycota fungal species have also been reported to have partially formed micro-channels present,^22^^,^^23^ although it is unclear whether these have any function. There are also no reports of any defined pathways or channels in the ECM layer of wood-degrading fungi that might permit channeling of extracellular enzymes. Several reports of filamentous structures observed in the ECM of *Postia* (*Rhodonia*) *placenta* and other wood-decay fungal species have been published but with no definitive information as to whether these filaments may play a role in metabolite transport.^24^^,^^25^^,^^26^^,^^27^

Relative to the size of metabolites which must pass through the ECM to promote substrate degradation, BR fungi have a unique decay mechanism where the wood cell wall is depolymerized via the action of low-molecular-weight (LMW) metabolites that generate powerful oxygen radicals, in a mechanism known as the chelator-mediated Fenton (CMF) mechanism.^8^^,^^28^^,^^29^^,^^30^^,^^31^^,^^32^ These LMW metabolites secreted by BR fungi and involved in cell wall degradation have been localized using transmission electron microscopy (TEM)-immunolabeling and have been shown to diffuse both within the ECM and also within the wood cell wall.^33^ It has previously been confirmed, and well established, that fungal enzymes are unable to penetrate intact plant cell walls,^33^^,^^34^^,^^35^^,^^36^^,^^37^ and as part of that research, immunolabeling assays and electron microscopy have also demonstrated that fungal extracellular enzymes are localized within the ECM.^11^^,^^24^^,^^33^ However, it has not been demonstrated whether high-molecular-weight enzymes have the ability to pass through the fungal ECM.

Wood-degrading fungi are known to secrete oxalate, and oxalate crystals are readily observed within the ECM of decay fungi both in culture and during wood degradation.^8^^,^^25^^,^^38^^,^^39^ Oxalate regulatory mechanisms employed by both WR and BR fungi are known,^40^^,^^41^^,^^42^^,^^43^ and metabolic flux analysis has been conducted in a BR fungus.^44^ Because of oxalate’s ability to sequester iron and in promoting or inhibiting CMF reactions,^45^^,^^46^ it is feasible that decay fungi would have the ability to regulate oxalate biosynthesis and secretion. Control of oxalate diffusion through the ECM also has not yet been explored; however, oxalate crystals in the ECM have been observed to be generated and then dissolved over time, and this has been proposed as evidence which demonstrates how the fungi may regulate soluble oxalate levels in the ECM.^38^^,^^39^

Considerable information is known about the chemistry of oxalate in relation to higher animal pathologies, and for example the role of oxalate and how it chemically interacts with proteins and tissue in kidney stone formation are well studied.^47^^,^^48^ Scant information is available however on the interaction of oxalate with polymers, with the exception of one limited report on the ability of oxalate to form hydrogels with chitin and chitosan gels in the food science literature^49^ and also research reports on the interaction of oxalate with cellulose nanocrystals.^50^ Given that oxalate has long been known to be present surrounding fungal hyphae, and proposed mechanisms involving oxalate in fungal degradation mechanisms have long been cited,^38^^,^^51^^,^^52^^,^^53^^,^^54^^,^^55^ surprisingly no information is available on the chemical interaction of oxalate with extracellularly secreted polymers from fungi such as β-1,3-glucan.

Only a handful of studies have examined the role of the ECM in fungal degradation, and no papers have explored how oxalate in the ECM may impact, and/or regulate, diffusion of metabolites into lignocellulose substrates and what conditions may mediate any such regulation. Because oxalate is known to be produced by, and is present within, the ECM of most decay fungi, examining the role of oxalate in fungal ECM formation and function was of interest. Specifically, this study is a first attempt to gain understanding of the role that oxalate and β-glucan in the ECM may play in mediating diffusion of important extracellular enzymes and metabolites, recognizing that only a simple ECM model is used. We examine the role that oxalate may have in interacting with β-glucan to produce a hydrogel that would be a physical requirement of a functional ECM. Hydrogel formation with β-glucan and oxalate has not previously been reported. We characterized the hydrogels produced and then further investigated the ability of oxalate to diffuse though those hydrogels, in addition to examining whether a protein that simulated extracellular enzymes would be capable of diffusion through the hydrogels. Finally, we characterized the hydrogel structure chemically using Raman spectroscopy and modeling using density functional theory^56^ (DFT) simulation of the structures and vibrational spectra. Laminaribiose, which contains a single beta 1,3-glycosidic bond, was used as a model compound for β-1,3-glucan in the simulations.

Note: Other publications have used the term extracellular polymeric substance (EPS) and related terms to describe the microbial ECM. These terms are valid, but we have chosen to use the ECM term in our work because this term is currently in wider use for both fungi and bacteria. The use of the term ECM also avoids the redundance of the word “substance” in the EPS term as a polymer, by definition, is already a substance.

## Results

### Rheometric properties of β-glucan

**Figure 1:**
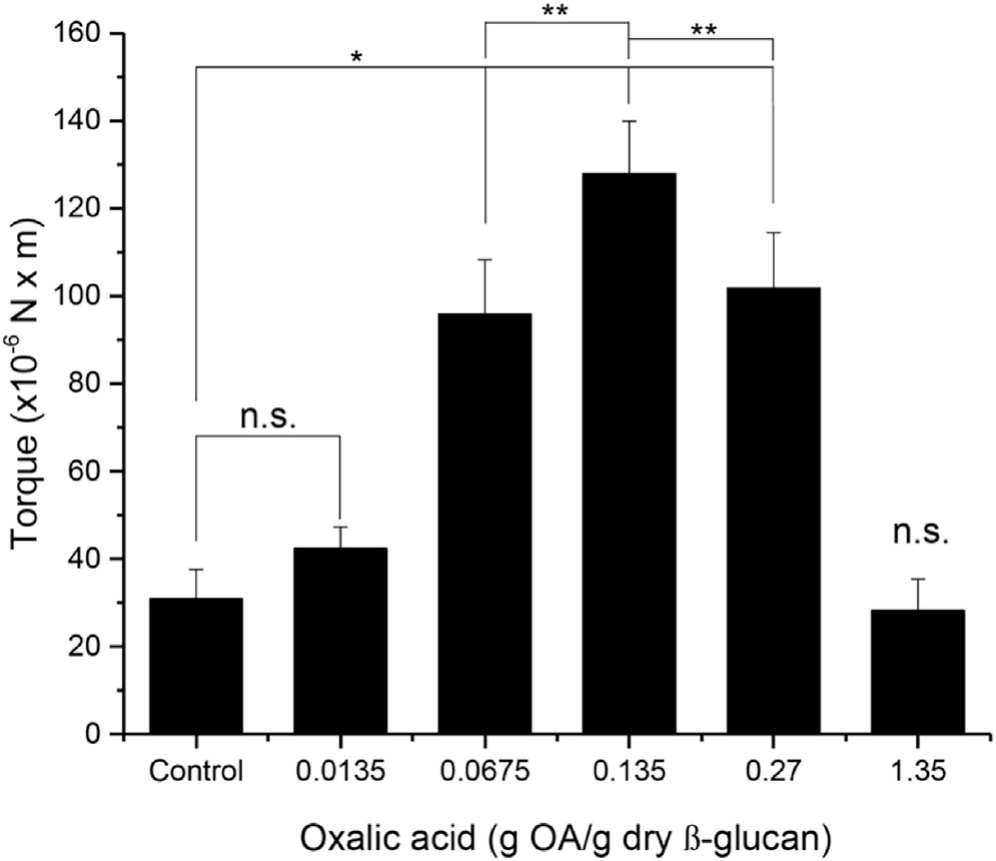
Torque at the fracture (yield) point of β-glucan gels in different oxalate concentrations Data as average ± std deviation, n = 5. n.s. not significant, ∗p < 0.0001, ∗∗p ≤ 0.01.

The formation of a β-glucan hydrogel with the addition of oxalate has not previously been reported; thus, in our initial experiments exploring how a generalized β-glucan ECM might be produced with oxalate addition, we were intrigued by the room-temperature formation of a hydrogel that increased in viscosity as oxalate was added. Rheometric analysis of defined β-glucan hydrogels with increasing amounts of oxalate demonstrated this increase (Figure 1), up to a ratio of 0.135 g oxalate/1.0 g dry β-glucan. Gels made at this optimal ratio were stiffer and exhibited a torque at the yield point that was significantly greater than that of control gels or at oxalate levels above or below this optimum (Figure 1).

### Hydration of β-glucan and retention of oxalate

**Figure 2:**
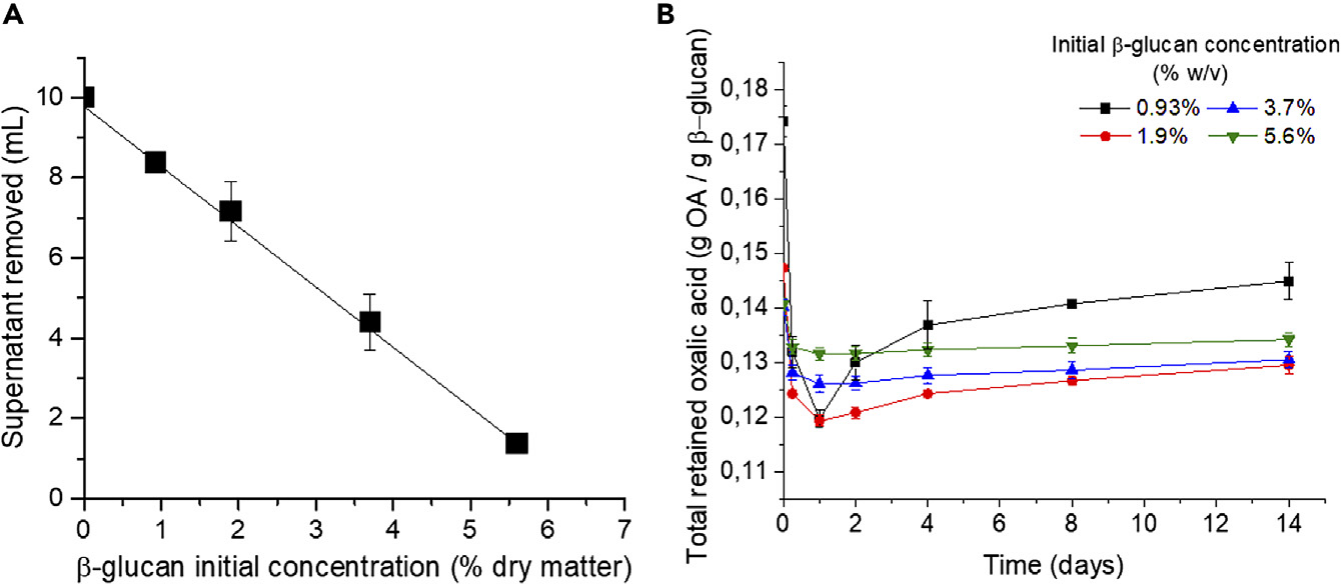
Water and oxalate retention by β-glucan (A) Supernatant removed from the top after gel formation. (B) Retention of oxalate (OA) by β-glucan gels. Initial concentration as % w/v dry β-glucan/ddH_2_O; data normalized by the amount of β-glucan (g). Presented as the average ±standard deviation, n = 3.

After gel formation, a supernatant layer was formed on top of the gels. The volume of supernatant was inversely proportional to the initial concentration of β-glucan (Figure 2A). The saturation point of β-glucan was at a concentration of 6.5% w/v, as calculated from extrapolation of the intercept in the x axis.

Initial oxalate concentration was measured in the supernatant and from the added layer of ddH_2_O. A greater initial retention of oxalate (0.17 g oxalate/g dry β-glucan) was observed in β-glucan gels prepared at 0.93 %w/v (initial β-glucan concentration) compared with gels prepared at 1.9, 3.7, and 5.6% w/v with a retention of oxalate of 0.15, 0.14, and 0.14 g oxalate/g dry β-glucan, respectively (Figure 2B, t = 0days).

In the 24 h following the addition of the layer of ddH_2_O, a significant decrease in retention of oxalate (p < 0.02) was observed as oxalate diffused out of the gels (Figure 2B, t = 1day). Gels made with a lower initial β-glucan concentration (0.93% w/v) lost up to 29% of the retained oxalate compared to the higher initial β-glucan concentrations (1.9, 3.7, and 5.6% w/v), where diffusional loss of oxalate was 8.5, 9.1, and 6.5%, correspondingly.

Over two weeks later, retention of oxalate by the gels significantly increased in gels prepared at 0.93, 1.9, and 3.7% w/v gels (p < 0.024), whereas it remained the same (p > 0.05) at an initial concentration of β-glucan of 5.6% w/v (Figure 2B). The retention of oxalate by β-glucan gels was calculated as 0.135 g oxalate/1.0 g dry β-glucan and this calculation was used in the following experiments accordingly.

### Diffusion of inorganic compounds and protein through β-glucan gels

The fungal ECM allows the diffusion of molecules secreted by the fungal hyphae. As a proxy for fungal ECM, the ability to diffuse small molecules 2,3-dihydroxy benzoic acid (2,3-DHBA) and protein (bovine serum albumin) was explored in β-glucan gels.

#### Diffusion of 2,3-DHBA through β-glucan gels

**Figure 3:**
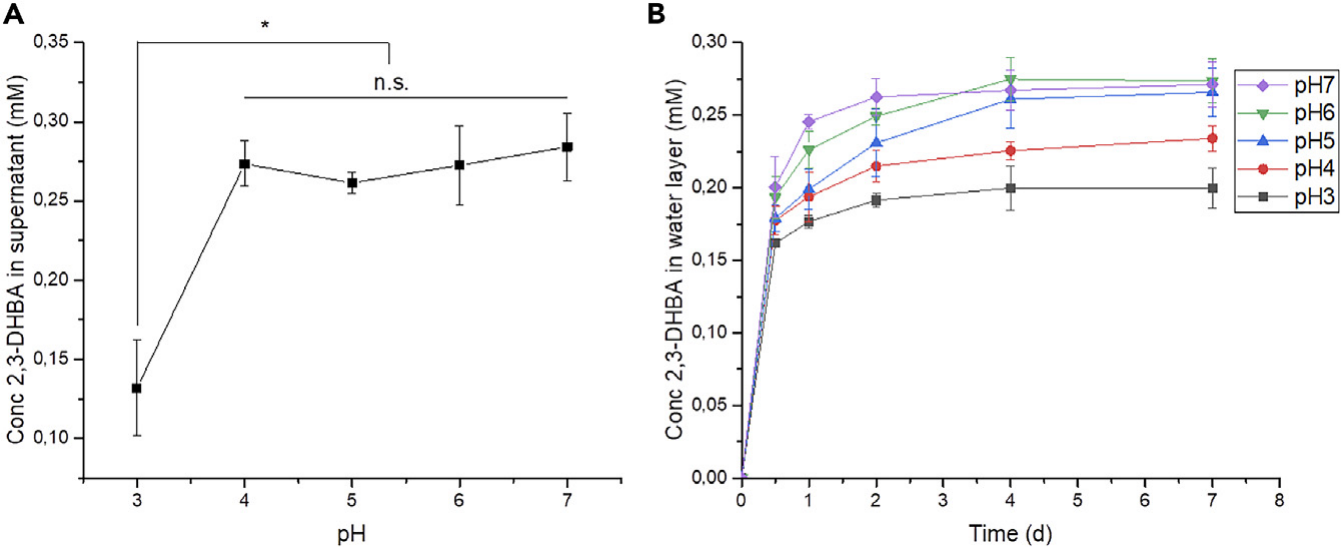
Diffusion of a model low molecular weight compound through ß-glucan-oxalate hydrogels Effect of the pH in (A) the concentration of 2,3-DHBA in the original supernatant layer on top of the β-glucan gel and (B) diffusion of 2,3-DHBA into the water layered on top of the β-glucan gel after the removal of the supernatant. Data presented as average ±standard deviation, n = 3. ∗p < 0.0001.

2,3-DHBA was measured in the supernatant and the ddH_2_O layer added after incubation. The concentration of 2,3-DHBA in the supernatant left after incubation was similar (∼0.28 mM) in gels adjusted to pH = 4.0–7.0, whereas it was significantly lower (0.13 mM) in gels adjusted to pH = 3.0 (Figure 3A). Despite the higher concentration of 2,3-DHBA retained by gels at pH 3.0, the diffusion of 2,3-DHBA into a fresh layer of ddH_2_O in these gels was significantly lower than that of gels at pH 4.0 (p < 0.05) and pH 5.0 and above (p > 0.001) (Figure 3B).

#### Diffusion of albumin through β-glucan gels

**Figure 4:**
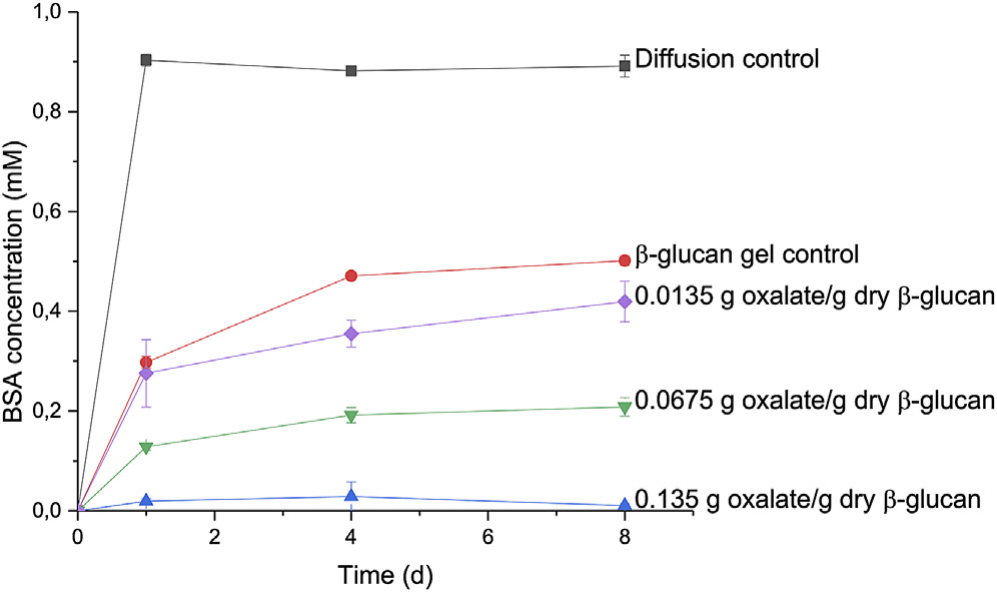
Diffusion of albumin (BSA) to the water layer after removal of the supernatant at pH 4.0 Data presented as average ±standard deviation, n = 3.

Albumin was measured in the supernatant and the ddH_2_O layer added after incubation. At pH 4.0, the diffusion of albumin through β-glucan control gels (Figure 4, red circle) was significantly reduced by 44% (p > 0.001) compared to free diffusion in a water column (Figure 4, black squares). Gels with added oxalate restricted the diffusion of albumin even further.

At low oxalate concentration (0.0135 g oxalate/g dry β-glucan), albumin diffusion was comparable to the control gels at 24 h (∼0.30 mM) and significantly lower (p > 0.01) after 8 days. Interestingly, an albumin retention of 77% and 99% was observed at higher concentration of oxalate (0.0675 and 0.135 g oxalate/1.0 g dry β-glucan, respectively), suggesting that albumin diffusion can be regulated by the concentration of oxalate in β-glucan gels.

### Chemical characterization of β-glucan gels with oxalic acid with comparative DFT and Raman spectroscopic analysis

#### DFT simulations of structure and bonding

To assess how oxalate interacts with β-glucan in gel formation, we examined the intermolecular interactions that occur between the two molecular structures. Because β-glucan is uncharged and both molecules consist of multiple hydrogen bond donating and accepting sites, the most likely gel-forming interaction is hydrogen bonding to form an oxalic acid-β-glucan complex.^57^ To elucidate the structure of the oxalic acid (oxalate ion) complex with β-glucan, we performed DFT simulations on combinations of oxalic acid and laminaribiose. Laminaribiose was chosen as the simplest “monomer” β-glucan model compound containing a β-1,3-glycosidic bond. Performing calculations on the model compound minimizes the calculation time required while still providing a relevant model structure for the interaction of the carbohydrate with oxalic acid. A benchmark was available in the form of simulations of β-1,3-glucan previously reported by Lee et al.^58^ who used 5 sugar (5mer) oligosaccharides as simple models for longer glucan chains. Furthermore, using the disaccharide minimized the complexity of the vibrational modes for characterization of the stimulated Raman spectra.

Simulations of laminaribiose included systematic combinations of oxalic acid (protonated and deprotonated H-oxalate and doubly deprotonated oxalate forms), with both single and two laminaribiose molecules to represent potential gel structures that might form at different pH values. The interaction of two laminaribiose molecules with a single oxalate (oxalic acid) molecule was chosen as representative of the gel structure based on comparison with similar structures previously reported in the literature.^49^^,^^50^ Hydrogen bonding between the oxalate protons and hydroxyl oxygens located on the carbohydrate was used as the initial structure, with several initial positions of the oxalate molecule selected to avoid conflating a local minimum with a global minimum. In all cases, optimized structures were insensitive to initial conditions, consistent with identification of a true global minimum.

**Figure 5:**
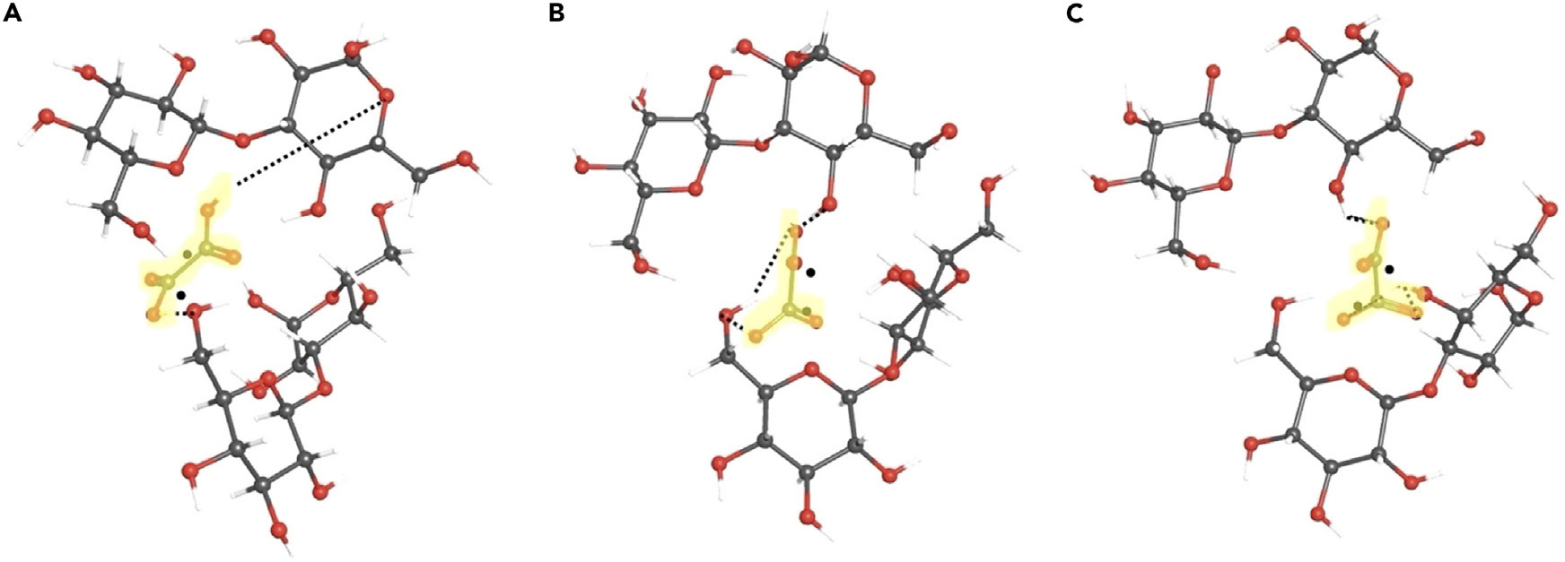
DFT-optimized simulation of a 2-sugar unit laminaribiose dimer (A–C) (2 sugar units) bonded by: (A) oxalic acid, (B) H-oxalate ion (−1), and (C) oxalate ion (−2). Oxalate molecules are highlighted in yellow. Oxalate carbon atoms (∗). H-bonds (dashed lines).

Optimized structures identified using DFT (Figure 5) for the laminaribiose-oxalic acid (Figure 5A) and laminaribiose-oxalate (Figures 5B and 5C) complexes all showed the formation of hydrogen bonds for any optimized hydrogen-to-oxygen separated by a distance between 1.6 and 1.8 Å. Oxalic acid, H-oxalate, and oxalate all formed hydrogen bonds that bridge between the two laminaribiose molecules. Interestingly, the hydroxyl groups of oxalic acid and H-oxalate donate hydrogen to participate in hydrogen bonding with laminaribiose, whereas the doubly deprotonated oxalate ion, which lacks a polar hydrogen, participates in hydrogen bonding by accepting a hydrogen from laminaribiose via its carbonyl group. The structure of the resulting molecular complex configures to accommodate different hydrogen bonding types, and the DFT-simulated structures demonstrate that complex formation will occur over a wide range of pH values from doubly deprotonated oxalate to its fully protonated form.

DFT provides thermodynamic estimates to help understand which forms of oxalate are most likely to form bridging complexes with β-glucan. Specifically, heats and free energies of formation of complexes involving laminaribiose are predicted to be very slightly negative (∼-0.05 kJ/mol) for both oxalic acid and H-oxalate (Figures 5A and 5B). The precision of this value is sufficient to indicate that the oxalic acid and H-oxalate will exist in an equilibrium with the bridged complexes to form gels. The corresponding values for doubly deprotonated oxalate (Figure 5C), in contrast, are positive 100 kJ/mol, values that indicate thermodynamic instability of oxalate/β-glucan complexes. As such, the thermodynamic parameter estimates predict the formation of complexes between β-glucan and oxalic acid and H-oxalate but not oxalate, indicating the importance of oxalic protons for forming stable bridges.

#### Raman spectroscopy

**Figure 6:**
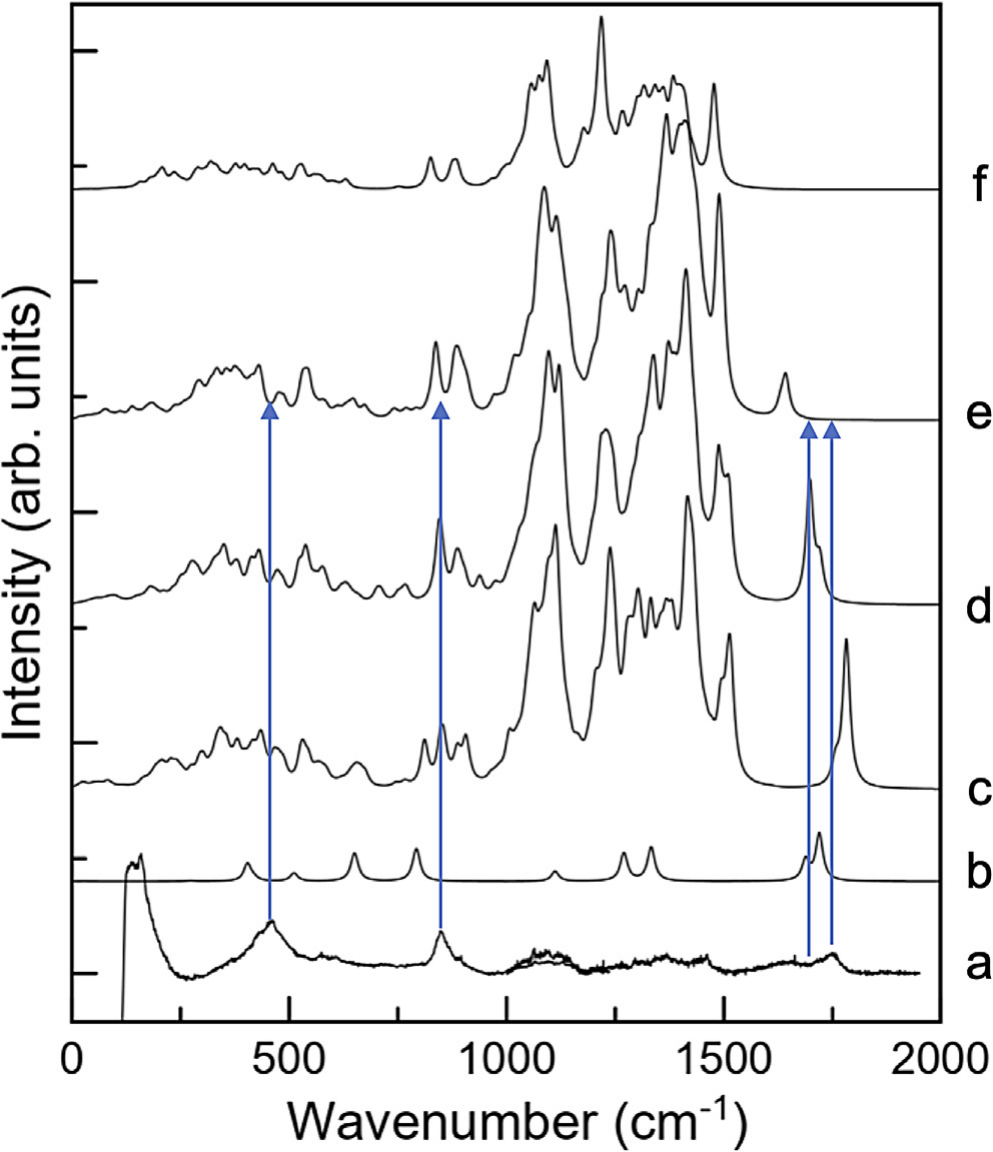
Experimental Raman spectrum of ß-glucan hydrogels compared to DFT simulated spectra of oxalic acid or laminaribiose with and without bridge oxalates The ß-glucan-oxalate hydrogel alone (a), compared with (b) DFT-simulated spectra obtained for the H-oxalate ion in water, (c) oxalic acid bridging two laminaribiose molecules, (d) H-oxalate (−1) bridging two laminaribiose molecules, (e) oxalate (−2) bridging two laminaribiose molecules, and (f) laminaribiose.

Because DFT analysis predicts at least two oxalate complexes can form either involving oxalic acid or H-oxalate, Raman spectroscopy was conducted to probe the structure of the hydrogels produced in this research. Raman spectroscopy is sensitive to protonation levels,^59^ hydrogen bond interactions,^60^ and complexation between acids and carbohydrates^61^ and therefore functions as an ideal tool to probe the molecular detail of hydrogel structure. Accordingly, Raman spectroscopy was used to explore the nature of the β-glucan/oxalate complex to determine which of the two plausible forms is more consistent with experimental spectra. Comparisons of DFT-simulated spectra and Raman spectra (Figure 6 and also Figures S1–S6) show the spectrum obtained for oxalic acid/β-glucan gel (Figure 6a) with several features at approximately 1750, 900, and 450 cm^−1^. Of these, the band at 1750 cm^−1^ is easily attributed to the carbonyl group in oxalic acid. The others arise either from glucan or oxalate/oxalic acid skeletal vibrations.

Spectra for oxalate (Figure 6b) and laminaribiose (Figure 6f) are compared with the DFT-simulated spectra derived in Figures 6c–6e, and this provides evidence of the likely form of oxalate in the β-glucan hydrogel. Focusing first on the carbonyl features, all three spectra simulated for oxalate-laminaribiose complexes contain a band at approximately 1750 cm^−1^ that is absent in the spectrum predicted for laminaribiose on its own. Accordingly, this feature is attributable solely to oxalate. Comparing the spectrum for the oxalic acid/β-glucan gel (Figure 6a) with the DFT-simulated spectra (Figures 6c–6e) indicates that the best match, in terms of position and especially shape, is the oxalic acid-laminaribiose complex (Figure 6c). The spectrum for the doubly deprotonated oxalate-laminaribiose complex (Figure 6e) is the poorest match as neither the shape of the band nor its location matches the experimental observation. The spectrum for the H-oxalate-laminaribiose complex (Figure 6d) matches the experimental position reasonably well, but the shape (consisting of a main band and a blue-shifted shoulder) does not match the experimental shape (consisting of a main band and a red-shifted shoulder).

Consideration of the carbonyl feature at ∼1750 cm^−1^ in the experimental and simulated Raman spectra supports the formation of complexes between oxalic acid and β-glucan in the gels formed in this study. That stated, the possibility of some mixture of oxalic acid and H-oxalate bridging groups exists. One final piece of evidence, the skeletal bands at 450 and 900 cm^−1^, were evaluated in an effort to remove any remaining ambiguity about the hydrogel structure. The 900 cm^−1^ band was present in any spectrum containing either β-glucan or laminaribiose indicating that this band has little diagnostic value pertinent to structural assessment. The feature at 450 cm^−1^ is broad for the experimental spectrum (Figure 6a) and for the simulated spectra corresponding to oxalic acid and H-oxalate complexes (Figures 6c and 6d, respectively). No broad feature is present in the spectrum simulated for the doubly deprotonated oxalate (Figure 6e), indicating that the ß-glucan-oxalate gels contained only negligible amounts of this species. Accordingly, consideration of the feature at 450 cm^−1^ again rules out a double deprotonated oxalate structure but is unable to differentiate between the presence of oxalic acid and H-oxalate in bonding to laminaribiose. This suggests that both bridging groups can exist in the β-glucan gel, likely in equilibrium as free molecules and in the complexes, and with the relative amounts depending on their concentrations and the pH of the gel. The Raman findings therefore explain robust hydrogel formation over a range of pH values as a consequence of different forms of oxalate serving as cross-linking groups at different pHs.

## Discussion and conclusions

The formation of β-glucan hydrogels with oxalate has not previously been reported; thus, our findings on the role of oxalate in β-glucan hydrogel formation are both novel and important in reference to microorganisms that produce ß-glucan-based ECMs and that also produce extracellular oxalate. Chitosan gels with oxalate have previously been reported in the food science literature, but those gels were formed only after heating, and data indicated that oxalate was bound to the chitosan in a non-covalent manner. The ability of β-glucan to form non-covalent oxalate hydrogels at room temperature where β-glucan binds oxalic acid and H-oxalate by hydrogen bonding is a unique finding important to biological systems because of the way in which oxalate concentration can potentially be altered within the hydrogel to change both the gel viscosity, as well as change the permeability to different fungal metabolites and substrate molecules.

Prior research considered that the fungal ECM’s physical properties arise largely based on the interaction of β–glucan or other carbohydrate polymers with structural proteins. In fungi that secrete oxalate, however, our research demonstrates that soluble oxalate will cross-link with β-glucan to form a hydrogel. A structure for oxalate interaction with β-glucan chains has been proposed in the current work with an equilibrium between two β-glucan/oxalate structures likely. We also found that control of soluble oxalate levels alters the physical properties of the β-glucan gels, allowing the viscosity of the hydrogels formed to be modified. This mechanism would potentially allow fungi to control, and even prevent, diffusion of large protein molecules. Further, hydrogen bonding of oxalate with β-glucan greatly limited diffusion of free oxalate, and this result suggests that diffusion of oxalate out of the fungal ECM into substrates would be limited. These results have implications for woody biomass degradation by BR decay fungi as the BR fungi employ CMF chemistry, which has been demonstrated to function based on the transfer of iron from a low-pH/high-oxalate concentration environment to a higher-pH/low-oxalate concentration environment in the wood cell wall. Oxalate diffusion limitation within, and from, the ECM as found in our current research is consistent with this mechanism. BR fungi also rely on LMW catecholate/hydroquinone/aminophenol chelators to bind iron, and the ability of these compounds to diffuse freely through the ECM is critical to CMF chemistry. Our data show that, although high-molecular-weight proteins would potentially be restricted from diffusing through the ECM, LMC hydroquinones would diffuse through the β-glucan/oxalate ECM model under all conditions tested.

Concentrations of oxalate within the fungal ECM have not been well studied. Previous researchers have used “squeezates” from BR decayed wood to try to approximate the total amount of soluble oxalate in the decayed wood, finding that concentrations ranged from 8.7 to 12.5 mM of soluble oxalate in decayed wood samples.^62^ However, this work did not account for the total amount of oxalate in BR decayed wood and ignored insoluble crystalline oxalate. Further, an unknown amount of soluble oxalate would remain bound to the fungal ECM, or within the disrupted lignocellulose cell walls, despite the use of high pressure to attempt to extract the oxalate. In addition, this work^62^ estimated the amount of oxalate within the wood, and not within the fungal ECM, which would be only a small fraction of the volume of the total mass of the sample being assayed. Other studies with Ascomycota fungi have found oxalate concentrations ranging from 20 to 35 mM in the media,^63^ indicating that concentrations within the fungal ECM would be higher. Our current research demonstrates that much of the oxalate produced by fungi remains bound within the β-glucan matrix even though some oxalate also likely diffuses within the wood cell wall. Therefore, we justify using oxalate concentrations in the 100 mM range as appropriate for this *in vitro* research on a synthetic ECM but recognize that concentrations higher and lower than this within the fungal ECM may exist depending on the physiological state of the fungus. Because oxalate crystals are often visualized in the fungal ECM,^64^ localized oxalate concentrations within the fungal ECM would necessarily reach saturation in these locations.

Relative to oxalate diffusion from the ECM into the wood cell wall, a recent paper by Füchtner et al.^65^ used confocal Raman microscopy to examine wood cell walls undergoing degradation by a BR fungus *Rhodonia placenta* and concluded that oxalate was present in the cell walls of the wood undergoing degradation. However, quantification of oxalate concentration in cell walls was not conducted in that work, and non-microbially degraded wood samples were not used for reference. Thus it was not possible to differentiate between oxalate accumulation caused by BR fungi and any natural oxalate present in the wood cell wall in that work. Our current research suggests that oxalate diffusion into wood cell walls undergoing BR degradation would be limited, but it does not preclude that some oxalate could be present within the wood cell walls. Our findings suggest only that oxalate concentrations must be higher within the fungal ECM than within the wood cell wall of BR degraded wood. An oxalate concentration-differential and pH-differential, between the fungal ECM and the wood cell wall, has been proposed previously^29^ to explain how iron transfers from oxalate to hydroquinone chelators during CMF decay chemistry. This in turn promotes Fenton chemistry and hydroxyl radical production within the wood cell wall for lignocellulose deconstruction and prevents those reactions from being generated in the ECM where damage to the fungal hyphae could occur. Iron-oxalate may diffuse more readily into wood cell walls from the fungal ECM compared to calcium oxalate or oxalate ions; however, we did not test that specifically in our current research, and study of the diffusion of other ions though the ECM is worth consideration in the future. CMF chemistry requires that at least some amount of iron-oxalate penetrates parts of the wood cell wall, and this would be consistent with both Füchtner et al. 2022^65^ as well as our current findings on the binding of oxalate by β-glucan in the fungal ECM.

Beyond fungal mechanisms, an enhanced understanding of the role of oxalate in the formation and structure of the ECM has implications for biodegradation and bioconversion processes. Even though filamentous fungi are the primary agents involved in efficient degradation of woody biomass in nature, most filamentous species have seen limited use in bioconversion processes. This is in part due to our lack of knowledge of how the fungal ECM is generated by these fungi and the role that the ECM plays in the delivery of extracellular enzymes and other metabolites to woody substrates. As an example, upregulation of specific fungal metabolites such as lignocellulose-degrading enzymes will have limited utility in applied applications if those enzymes cannot also diffuse through the ECM to reach the substrates. Altering expression of pathways involved in oxalate production^44^ and secretion may be required to promote transport of enzymes through the ECM. Alternately, exploration of chemistries that can modify the permeability of the ECM through manipulation of soluble oxalate concentration in bioreactors may be required to effectively use some types of filamentous fungi effectively in bioprocessing operations and make the most effective use of both their LMW degradative metabolites as well as their degradative enzymes. Better understanding of both fungal enzyme and LMW metabolite passage into wood substrates will also allow refined wood degradation models to be generated, thus providing data for improved carbon cycling and climate change models where currently fungal degradation data are lacking.^66^^,^^67^^,^^68^

Finally, although this research is focused on environmental microbiology, it has potential implications for fungi and bacteria in other systems as well. Oxalate and other LMW organic acids are produced by many other fungi, and these acids could also potentially interact with the structural carbohydrates of the ECM in fungi important in biomedical pathologies. Alteration of fungal, and potentially bacterial, ECMs as part of microbial biofilms should be explored in future work to determine how these acids may interact with β–glucan and other structural carbohydrates in the extracellular polymeric structures of these fungi and how this may impact secretion of metabolites involved in pathogenesis. Previous research has noted that the fungal ECM serves as a barrier to antifungal treatments of human and animal disease.^13^^,^^69^ Thus, our current research suggests that regulation of the diffusional properties of the ECM by disrupting oxalate biosynthesis (or similar LMW cross-linking organic acids in other fungi) may be necessary to improve therapeutic penetration of the fungal ECM by certain antifungals.

### Limitations of the study

This study simulates the chemistry that is employed by fungal systems, but because of the complexity of fungal filamentous systems and the need to focus specifically on the components of the ECM, fungi were not utilized in this work. Instead, prior research on fungal systems is relied upon to provide background on the basic chemical components of the ECM that were used in this research. Thus, other minor components of the ECM (more limited carbohydrates and proteins) might interact to a limited extent in ways not recognized relative to hydrogel formation and diffusional properties. Prior research has demonstrated that β-glucan is the predominant carbohydrate in the fungal ECM; however, β-glucan is a variable polymer with different degrees of branching. We used fungal (*Saccharomyces*) 1,3/1,6 β-glucan, but the degree of 1,6 branching is known to vary with fungal species, and this is a recognized limitation of our research. Although replicate studies were performed, only a single protein (bovine serum albumin) was used in diffusional studies. Although other proteins could potentially behave differently, our focus was in exploring size/mass-related diffusional properties and BSA was utilized as our chosen model.

## STAR★Methods

### Key resources table

**Table:** 

REAGENT or RESOURCE	SOURCE	IDENTIFIER
**Chemicals**		

Oxalate	Acros organics, NJ, USA	https://www.fishersci.com/shop/products/oxalic-acid-98-anhydrous-thermo-scientific/AC186432500
ß-glucan 80% from *Saccharomyces cerevisiae*.	PureBulk, Inc. OR, USA	https://purebulk.com/products/beta-glucan-1-3-1-6-80
Bovine Serum Albumin: fraction V, fatty acid free. 66.4 g/mol	MilliporeSigma, St. Louis MO, USA	https://www.sigmaaldrich.com/US/en/product/sigma/a9576?gclid=CjwKCAjwzuqgBhAcEiwAdj5dRhcFXc_u16HKadxSel4RoZGNzIoiqkjpc7DdJIlq6d_ALec3joxZ0BoCepMQAvD_BwE&gclsrc=aw.ds
Bradford assay kit	Sigma-Aldrich, MO, USA	https://www.sigmaaldrich.com/US/en/product/mm/2740op
2,3-dihydroxybenzoic acid	MilliporeSigma, St. Louis MO, USA	https://www.sigmaaldrich.com/US/en/product/aldrich/126209
Gold nanoparticle suspension (20 nm, OD 1, in citrate buffer)	Sigma Aldrich, MO USA	https://www.sigmaaldrich.com/US/en/product/aldrich/741965

**Software and algorithms**		

Gaussian 09 Software package	Gaussian Inc. Wallingford, CT. USA	https://gaussian.com/wp-content/uploads/dl/us_acad.pdf
WebMO	Enterprise WebMO LLC. Tucson, Arizona. USA	http://www.webmo.net
OriginPro® 2020	OriginLab, Northampton, Massachusetts. USA	https://www.originlab.com/2020
MagicPlot student version 2.9.3 software	Magicplot Systems, LLC	https://magicplot.com

### Resource availability

#### Lead contact

Further information and requests for resources and reagents should be directed to and will be fulfilled by the corresponding authors: Barry Goodell (bgoodell@umass.edu - lead contact) and Geoffrey Tompsett (gtompsett@wpi.edu).

#### Materials availability

This study did not generate any new unique reagents or materials to report. All reagents or materials used are commercially available.

### Method details

#### Oxalate retention and diffusion in β-glucan/oxalate gels

To determine the extent of oxalate mobility once β-glucan/oxalate gels had formed, diffusion studies were performed to assess how much oxalate would diffuse through β-glucan/oxalate gels made with different concentrations of β-glucan. β-glucan/oxalate gels were prepared in Falcon tubes by dispersing commercially available 1,3/1,6 β-glucan (80% from *Saccharomyces cerevisiae*. PureBulk, Inc. OR, USA) in oxalate solution (Acros organics, NJ, USA). The tubes were then incubated (24h, 37°C) to permit gel formation.

To determine the retention of oxalate, in separate experiments, β-glucan (0.93, 1.9, 3.7, and 5.6% dry w/v) was dispersed in oxalate (10 mL, 100 mM) with deionized distilled water (ddH_2_O - 18.2MΩ∗cm) used as control. After incubation any supernatant was removed, and the volume and concentration of oxalate determined to correct the oxalate concentration in the gels. A ddH_2_O (1 mL) layer was then added to the top of each gel and the tubes incubated again (37°C) to allow any diffusion of oxalate from the gel into the water layer. Gels were made in triplicate with the water layer in each tube sampled 6 times (100 μl aliquots) over 14 d.

Oxalate was quantified in both the supernatant and the added water layer using liquid chromatography - HPLC (Shimadzu Co. Japan); Column: Aminex HPX-87H ion exclusion, mobile phase: isocratic H_2_SO_4_ 0.005 M, oven: 20°C, flow rate: 0.5 mL/min, detection: UV - 205 nm. A standard curve (linear range: 0.01 – 20 mM, r^2^=1.00) was constructed with the samples diluted appropriately as needed to fit the linear range.

#### Determination of viscosity of β-glucan gels by yield rheometry

β-glucan gels and controls were prepared as described above except that the β-glucan concentration was fixed (6.5% w/v) and, in separate experiments, different concentrations of oxalate were used (40 mL, 0.0135, 0.0675, 0.135, 0.27, and 1.35 g oxalate/g dry β-glucan). After incubation (24h, 37°C) in 50ml Falcon tubes the samples were brought to room temperature (20°C) and any supernatant was again removed for analysis. Five replicates were used for each condition.

Yield rheometry was conducted (Brookfield YR-1, MA, USA) to determine how oxalate affected β-glucan gel formation. The Brookfield spindle (V-73, vane length= 2.535 cm; vane diameter= 1.267 cm) was inserted at the primary immersion mark (yield multiplier constant= 10.0). Parameters: speed: 0.6 rpm, increment: 167 msec (1/6^th^ second), torque reduction: 100%.

#### Diffusion of 2,3-dihydroxy benzoic acid (DHBA) through β-glucan gels

The catechol-derivate compound: 2,3-dihydroxybenzoic acid (2,3-DHBA) was used to investigate the diffusion of small molecules through the fungal extracellular matrix (ECM). Catechol-derivates are known to be secreted by species of brown rot fungi and play a role in extracellular wood degradation mechanisms. Gels between pH 3.0 and 7.0 were made to evaluate the effect of the ionization of oxalate (pKa_1_=1.27; pKa_2_=4.28) in the diffusion of 2,3-DHBA (pKa=2.91).

Hydrogels were prepared as described above (10 mL, 6.5%w/v β-glucan, 0.135 g oxalate/1.0 g dry β-glucan). The pH was adjusted to 3, 4, 5, 6, and 7, by adding either hydrochloric acid or sodium hydroxide prior to incubation (24 h, 37°C). The supernatant was then removed and fresh ddH_2_O (1 mL) was layered on top prior to a second incubation (7 d, 37°C). The concentration of 2,3-DHBA was measured in the supernatant and samples of the fresh ddH_2_O layer (5x100 μl) by HPLC (Column: Supelco C-18; Mobile phase: Acetonitrile:water (1:1); Flow rate: 0.5 mL; Detection: UV at 280nm). Gels were made in triplicate.

#### Diffusion of albumin through β-glucan gels

Albumin (bovine serum albumin, BSA fraction V, fatty acid free. 66.4 g/mol) was used as a model protein as a proxy for extracellular fungal protein metabolites in the fungal ECM to explore the extent which proteins might be expected to diffuse within simulated β-glucan/oxalate ECM hydrogels. Gels were made in a gradient of oxalate to evaluate the effect of the stiffness of the gel in the diffusion of albumin.

Hydrogels similar to those described above were prepared with added albumin. β-glucan (6.5% w/v) was mixed with 10mL of albumin (1 g/L) and oxalate (0.0135, 0.0675, and 0.135 g oxalate/1.0 g dry β-glucan). Albumin without oxalate, and albumin without oxalate and β-glucan, were used as controls. After incubation (24 h, 37°C), the supernatant was removed and analyzed as above. A layer of double distilled H_2_O (1mL) was added above the gel and the tubes incubated again (14 d, 37°C). The water layer was the sampled (100 μl/sample) 4⨱ over 14 d. Samples were stored (-20°C) for protein quantitation with albumin quantified using a standard Bradford assay (Sigma-Aldrich, MO, USA) at 595 nm. Gels were made in triplicate.

#### Raman microscopy

Raman spectra of the β-glucan/oxalate gels were obtained using a Horiba XploRa Raman microscope operating with a 785 nm laser line at a power of ∼30 mW. A 2 s scan time was used with an accumulation of at least 40 scans. A 1200-line grating was used with an aperture of 100 and slit width of 300. Laser light was focused on the sample using a 100⨱ magnification lens with an Olympus microscope. 6.5% β-glucan gels (pH 4.0) with various concentrations of oxalate were made using a gold nanoparticle suspension (20 nm, OD 1, in citrate buffer, Sigma Aldrich, USA) to improve signal/noise ratio. Gel was smeared on either a glass microscope slide or a gold-coated silicon wafer and covered with a quartz cover slide to minimize drying. Alternatively, gels smeared on glass slides were air-dried at room temperature before Raman spectra were obtained.

#### Density functional theory (DFT) parameters and simulations

All DFT calculations were performed using a Gaussian 09 Software package^70^ and the WebMO interface.^71^ Geometry optimizations and frequency calculations were performed using Becke's three parameter Lee-Yang-Parr (B3LYP) hybrid functional and the 6-31G(d) routine basis set.^72^ Calculated band locations were corrected by multiplication by an empirical constant (0.9813) to improve accuracy, following the recommendation of Merrick et al.^73^ An implicit solvent (water) was selected for all calculations. Simulated spectra were generated by calculating Raman vibrational intensities and applying Gaussian broadening to the vibrational peaks of 10 cm^-1^. Visual identification of the individual modes was used to determine the molecular vibration responsible for each peak.

Laminaribiose was chosen as the simple representative of a carbohydrate possessing the β-1,3-glycosidic bond, and was chosen to minimize the calculation time required while providing a model structure for the interaction of carbohydrate with oxalate. Simulations of the Raman spectra of β-1,3-glucan were previously reported by Lee et al.^58^ who used 5 sugar (5mer) oligosaccharides as simple models for longer glucan chains. Molecules were constructed using the WebMO interface of laminaribiose with oxalate non-bonded and bonded to different carbons on the sugar molecules. Similarly, the bonding of two laminaribiose molecules and a single oxalate group were geometrically optimized in different configurations.

### Quantification and statistical analysis

Analysis of variance (ANOVA) and post-test Tukey analyses were performed to determine the significance of the comparisons between means using OriginPro® 2020 (OriginLab) software.

## Data Availability

• All data reported in this paper will be shared by the lead contact upon request. • This paper does not report original code. • Any additional information required to reanalyze the data reported in this paper is available from the lead contact upon request.

## Supplemental information

Document S1. Figures S1–S6

Figure360. An Author Presentation of Graphical Abstract
