# Supplementary material for: Interaction of oxalate with β-glucan: Implications for the fungal extracellular matrix, and metabolite transport
Source: iScience. 2023 May 10;26(6):106851. doi: 10.1016/j.isci.2023.106851 (PMC10232728; doi:10.1016/j.isci.2023.106851)
Supplement: Document S1. Figures S1–S6 [file mmc1.pdf]

iScience, Volume *26*

Supplemental information

Interaction of oxalate with β-glucan: Implications for the fungal extracellular matrix, and metabolite transport

Gabriel Perez-Gonzalez, Geoffrey A. Tompsett, Kyle Mastalerz, Michael T. Timko, and Barry Goodell

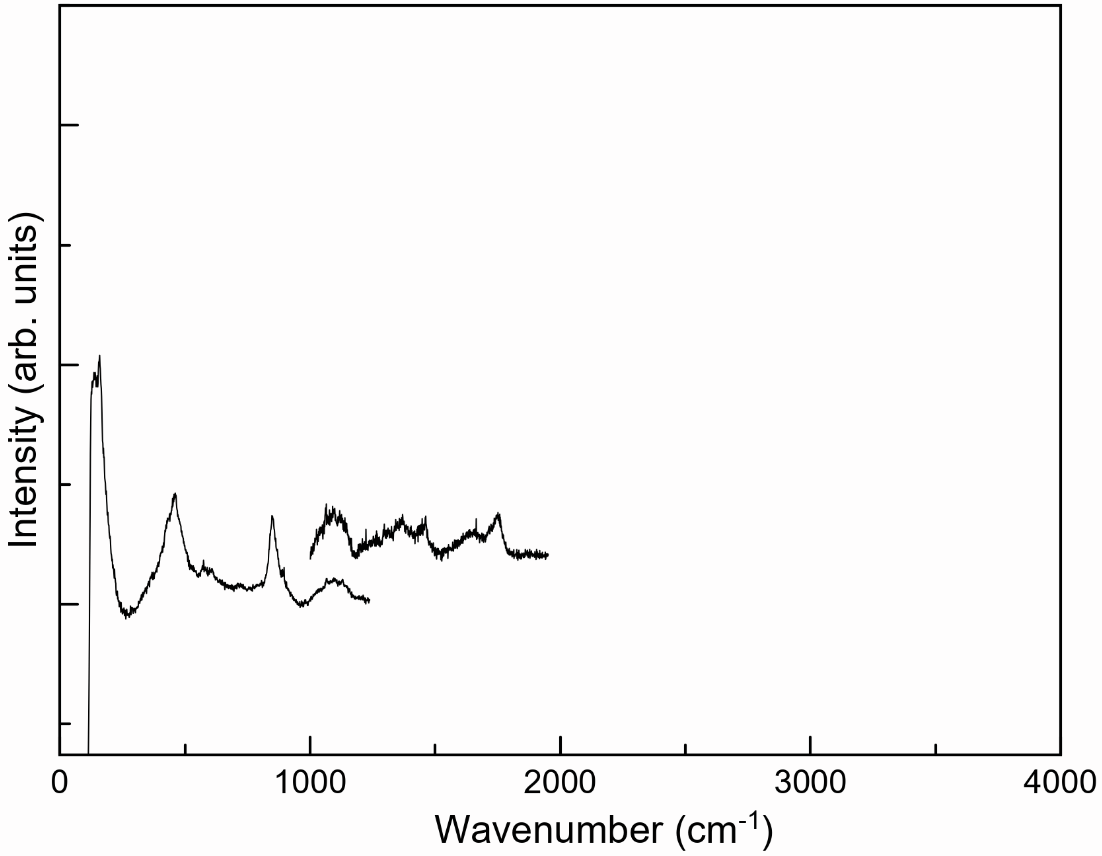

Figure S1 Experimental Raman spectra of 8%glucan with 0.5M oxalic acid gel. 785 nm, 1200 grating,~30 mW, 2 s scan time for 40 scans. Full spectrum detail of the spectrum in Figure 6.

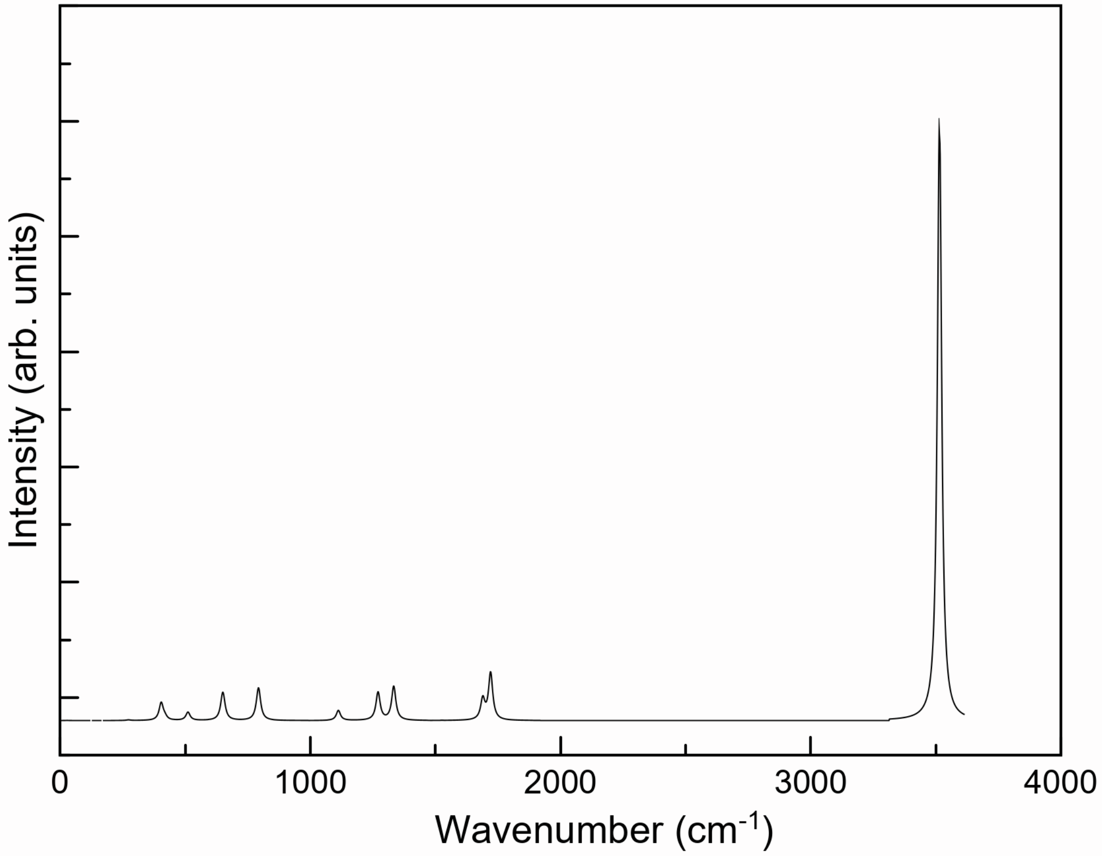

Figure S2 DFT simulated Raman spectrum of oxalic acid in implicit water solvent. Gaussian 09 Software package and the WebMO interface. Geometry optimizations with (B3LYP) hybrid functional and the 6-31G(d) routine basis set. Full spectrum detail of the spectrum in Figure 6.

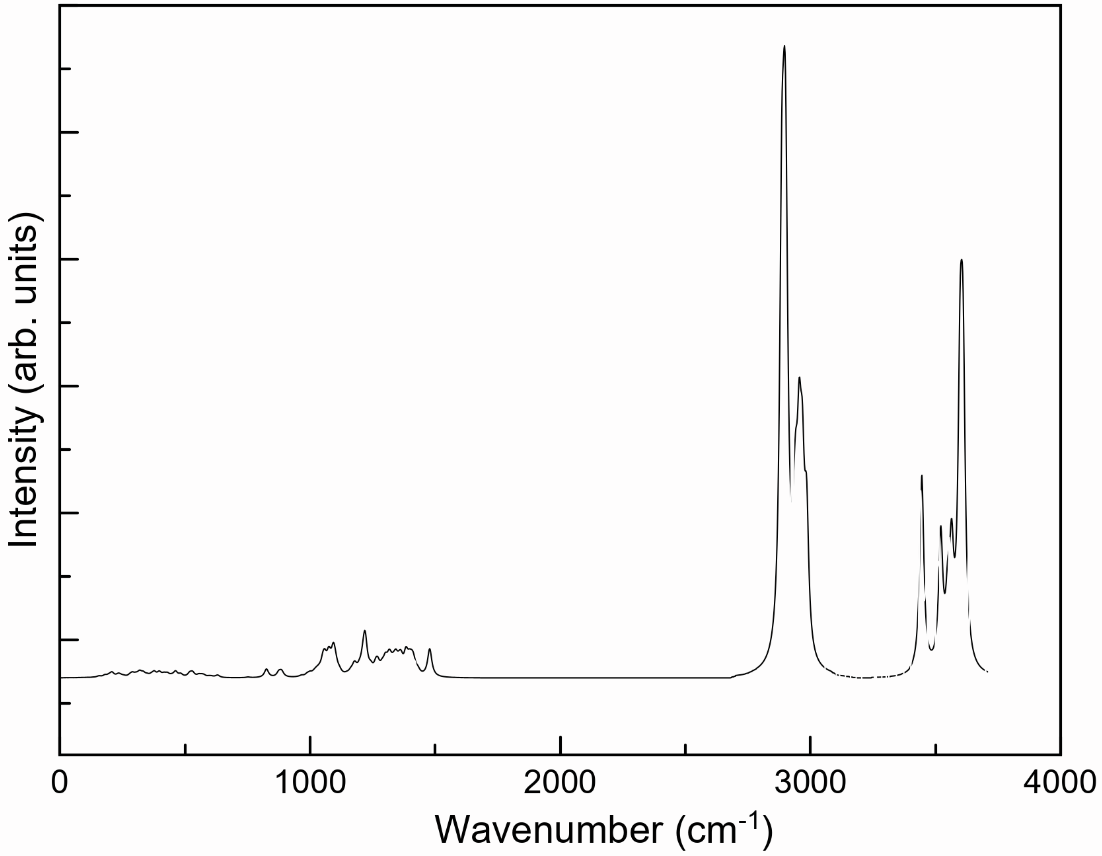

Figure S3 DFT simulated Raman spectrum of laminaribiose in implicit water solvent. Gaussian 09 Software package and the WebMO interface. Geometry optimizations with (B3LYP) hybrid functional and the 6-31G(d) routine basis set. Full spectrum detail of the spectrum in Figure 6.

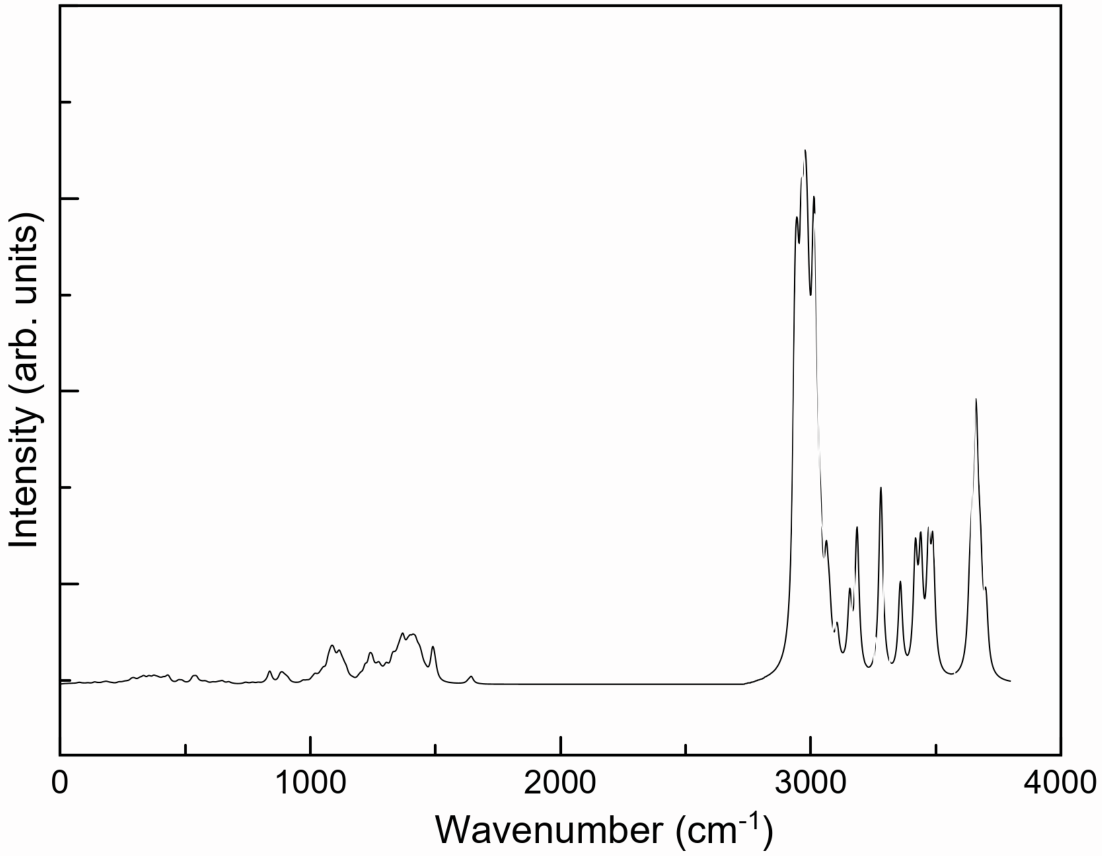

Figure S4 DFT simulated Raman spectrum of two laminaribiose molecule hydrogen bonded to oxalate ion in implicit water solvent. Gaussian 09 Software package and the WebMO interface. Geometry optimizations with (B3LYP) hybrid functional and the 6-31G(d) routine basis set Full spectrum detail of the spectrum in Figure 6.

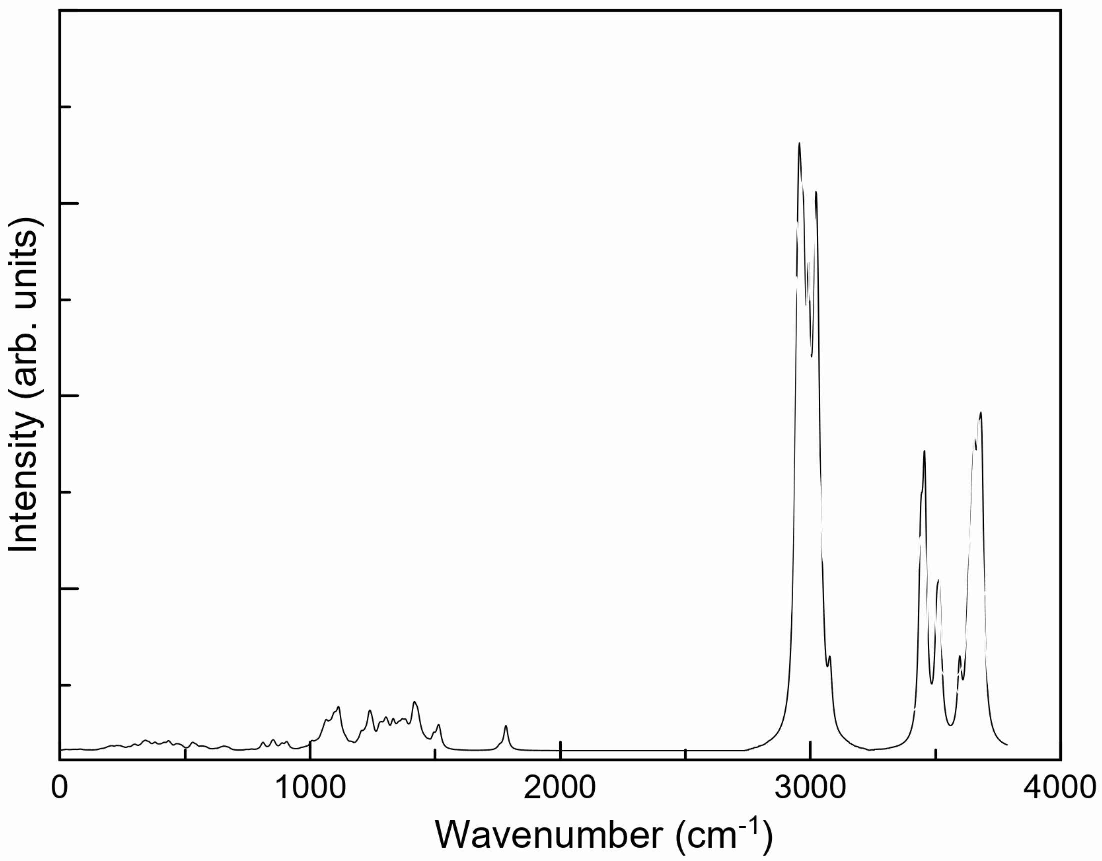

Figure S5 DFT simulated Raman spectrum of two laminaribiose molecules hydrogen bonded to oxalic acid in implicit water solvent. Gaussian 09 Software package and the WebMO interface. Geometry optimizations with (B3LYP) hybrid functional and the 6-31G(d) routine basis set. Full spectrum detail of the spectrum in Figure 6.

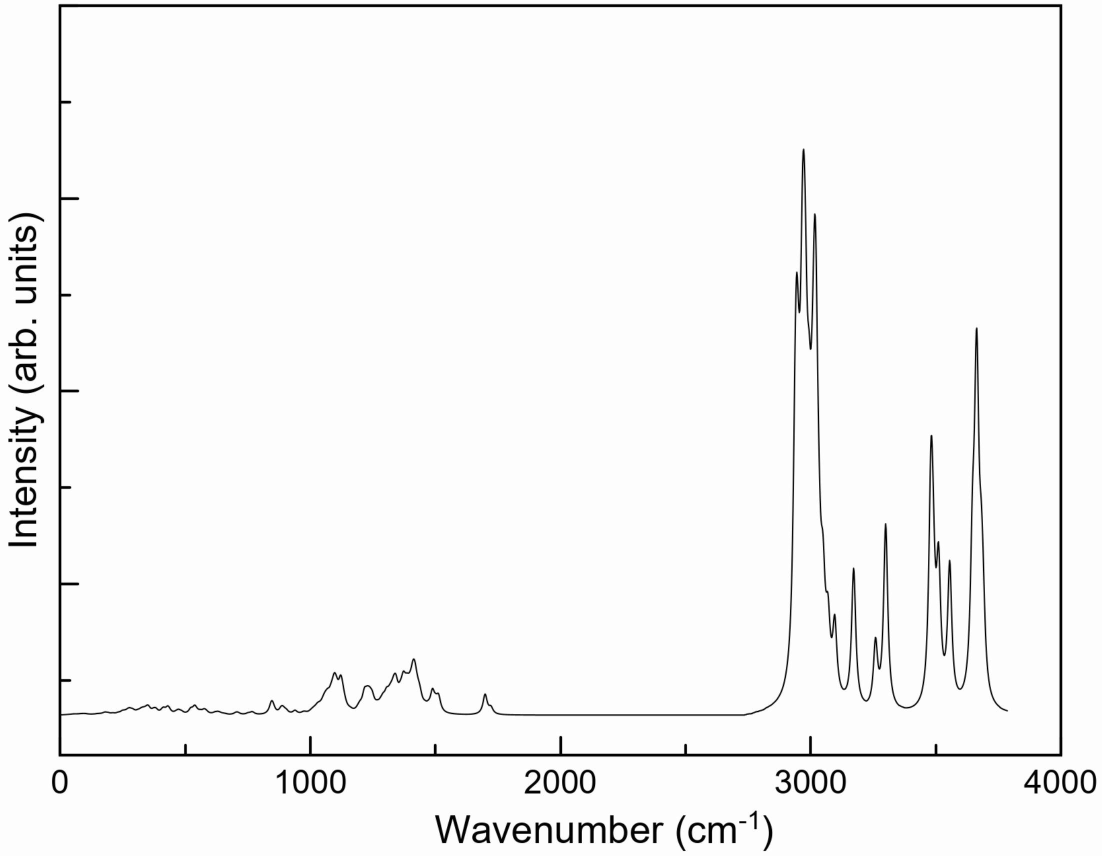

Figure S6 DFT simulated Raman spectrum of two laminaribiose molecules hydrogen bonded to hydrogen-oxalate ion in implicit water solvent. Gaussian 09 Software package and the WebMO interface. Geometry optimizations with (B3LYP) hybrid functional and the 6-31G(d) routine basis set. Full spectrum detail of the spectrum in Figure 6.
